# Driver models for the definition of safety requirements of automated vehicles in international regulations. Application to motorway driving conditions

**DOI:** 10.1016/j.aap.2022.106743

**Published:** 2022-09

**Authors:** Konstantinos Mattas, Giovanni Albano, Riccardo Donà, Maria Christina Galassi, Ricardo Suarez-Bertoa, Sandor Vass, Biagio Ciuffo

**Affiliations:** European Commission Joint Research Centre, Ispra, Ispra, Italy

**Keywords:** Connected and automated vehicles, Driver model, Safety requirements, Fuzzy logic

## Abstract

UN Regulation 157, the first global regulation regarding the type-approval of Automated Driving Systems (ADS), has been adopted in 2021. In it, safety performance requirements are being defined for vehicles of automation Level 3, according to the SAE J3016, with a limited Operational Design Domain (ODD). In particular, for three types of events that are related to motorway driving, two models are provided to distinguish between preventable traffic scenarios, for which the ADS is expected to avoid an accident, and unpreventable traffic scenarios, for which accidents cannot be avoided and the ADS can only mitigate their severity. The models recreate the short-term behavior of a driver who reacts to an emergency. Two possible actions are predicted: either no reaction or full braking when danger is identified. In the present paper the two models are analyzed and compared with two additional models: an industry proposed model, the Responsibility Sensitive Safety framework (RSS), and the Fuzzy Safety Model (FSM) proposed by the authors. As in the case of the two regulation models, also the RSS, although more sophisticated, assumes that the possible reaction by the driver is binary. This approach neglects the ability of a human driver to drive defensively and anticipate possible risks. Defensive drivers, indeed, may use comfortable decelerations in anticipation, to avoid finding themselves in an emergency situation. The FSM uses fuzzy logic to mimic this behavior.

Results show that anticipation plays a very important role to reduce the number of unpreventable traffic scenarios. In addition, by validating the classification capabilities of the four models with real traffic data, the FSM proved to be the most suitable of the investigated models. On the basis of these results, the FSM has been included in the proposal for amending UN Regulation 157, thus allowing to set higher safety standards for the first automated vehicles that will be introduced into the market.

## Highlights

• The safety of future automated vehicles depends on the requirements defined by international regulations.
• Safety requirements should be testable, verifiable and robust.
• Safety requirements should not dictate how an automated vehicles should operate but set their performance.
• Proper models should be used to set performance requirements to reproduce the complexity of a traffic scenario.
• Fuzzy logic is an appropriate tool to define the performance requirement of automated vehicles.

## 1 Introduction

In 2021, the first global regulation on the approval of automated driving systems (ADSs) has been adopted (UNECE, 2021). The UN Regulation 157 concerns the approval of a system able to drive without the strict supervision of a human driver in a very limited operational design domain (ODD), namely on motorways up to a maximum speed of 60 km/h (given lane changing is not possible, the system is also referred to as the Automated Lane Keeping System, ALKS). Since a driver is always required to be present to take-over the driving task, this is a Level 3 automated system according to the SAE classification (SAE, 2021). The limited and very well-defined scope of the Regulation helped to shape a legislation on the approval of ADSs in a reasonable timeframe and allowed to understand the issues to be tackled in its extension to wider and more complex scopes. It can be therefore considered a crucial milestone in the future legislative development in this field.

One of the most challenging issues the regulators had to address was the definition of the Level of safety the new vehicles have to guarantee and the way to prove it by the ADS developer. In principle, ADSs should i) respect traffic rules, ii) be able not to cause any accident, and, to the extent possible, iii) prevent accidents caused by other road users. It is easy to understand that turning this principles into quantitative requirements that can be proven by ADS developers during the type-approval process is not a simple task (Kalra and Paddock, 2016).

In general, four different approaches could be followed by the regulator (own elaborations based on Blumenthal et al., 2020):

- a first approach would set an overall safety target (in terms of e.g. accident probability, number of injured people, number of deaths, etc.) in the ADS’ ODD and leave the ADS developer to provide convincing evidence that this is achieved;
- a second approach would set a safety target on a fixed number of traffic scenarios and witness that the ADS is able to achieve it on the basis of test results in all of them (in which the results are weighted by the probability of occurrence of each scenario);
- a third approach would be not to set any safety target but to set some performance requirement allowing to define the traffic scenarios that an ADS should be able to safely handle without incurring into an accident; and finally
- a fourth approach setting operational requirements (namely requirements on the way the ADS should operate in specific circumstances) can also be considered.

The different approaches have all advantages and disadvantages. The first is, for example, the desirable approach for policy makers who can directly turn the policy objectives on road safety into a requirement for ADSs, but it is also very difficult to prove its fulfillment by an ADS developer. The third, on the contrary, is the desirable approach for ADS developers and Approval Authorities. It indeed clearly sets the vehicle design target for the formers and it can be assessed by the latter in a fairly straightforward way. However, this approach does not allow to directly understand the impact of ADS introduction on road safety as the performance requirements say little on e.g. the number of accidents that by respecting them an ADS is able to avoid compared to a human driver. Consequently, if these targets are not properly set or are set in a completely arbitrary way, the regulation may turn into a “procrustean bed^1^” with potentially harmful side effects. The risk of falling into a *procrustean bed* is even higher with the fourth approach. By arbitrarily limiting the design space of ADS manufacturers, indeed, there is a high risk to produce sub-optimal driving logics with possible negative effects on the safety of ADSs and on other dimensions such as, for example, the traffic flow. For these cases, it is therefore crucial that the requirements are based on strong empirical evidence and try to be as less arbitrary as possible.

UN Regulation 157 mainly adopts the third approach, without an overall safety target and with a series of performance requirements (complemented by a limited number of operational requirements on the need to respect traffic rules and on the minimum time headway to maintain from the vehicle in front). In the attempt to be as less arbitrary as possible, these requirements are based on physical considerations, such as the minimum braking distance of a vehicle, the minimum reaction time of their sensing systems, a quantitative definition of a competent and careful human driver, all to be used as a reference for the performances of future ADSs. This approach was in line with what had been used in the past for systems with lower levels of automation and was justified by the limited scope of this first regulation, which, in the near future, would therefore not be able to produce harmful effects neither to the safety nor to the efficiency of traffic flow.

As new regulations are currently being developed at UN and national levels it is however important to have a closer look at the implications of the aforementioned requirements and, in particular, if other approaches than those currently used could contribute to make them more robust. The attractiveness of using performance requirements is indeed undeniable, as they make the type-approval process transparent and easily verifiable. However, they are not easy to be properly set, especially when the ADS complexity increases. Therefore, it is important to understand whether they are a suitable tool or not.

The present paper tries to contribute to answering this question. It focuses on the type of performance requirements defined in UN Regulation 157. They are analytical models acting as classifiers, as they are used to define whether a specific traffic scenario can be considered preventable or not by the ADS. Such requirements are compared to other frameworks recently proposed in the literature such as the Responsibility Sensitive Safety (RSS) model, an industry-developed framework, and the Fuzzy Safety Model (FSM) proposed by the authors. Fuzzy logic has been introduced by Zadeh (Zadeh, 1965), and has been widely used in multiple disciples. In a previous work, the use of fuzzy logic to evaluate car-following safety levels has been investigated (Mattas et al., 2020). The relevant fuzzy safety metrics are exploited in this work to design the FSM, a driver reaction model. In this way, a scheme to extract performance requirements out of safety metrics is presented. All the models are then compared and validated against human driver data from the openly available highD dataset (Krajewski et al., 2018), to understand whether they are suitable to fulfill their task. It is shown that, while the current performance requirements in UN Regulation 157 are a step forward in establishing reasonable and transparent rules, in some cases they neglect the ability of drivers to prevent an accident by driving defensively. FSM is shown to be capable of reproducing this capability by using mild actions earlier and to avoid the need for harsh and imminent reactions later.

The paper is organized as follows. Section 2 introduces the relevant literature in the field. Section 3 includes the details of the four different models. The methodology used for the assessment is presented in section 4. Finally, results and conclusions are discussed in sections 5 and 6 respectively.

## 2 Related work

Connected and automated mobility is considered an important tool to achieve different policy objectives. In Europe, for example, they are expected to support the transition to a safer, more efficient, and more sustainable transport system (Marques dos Santos et al., 2022). Ensuring that automated vehicles are deployed with sufficiently ambitious safety requirements should be therefore a policy assumption. Furthermore, achieving high safety standards is very important for the public acceptance of automated vehicles, even regarding automated public transport (Kassens-Noor et al., 2020). Researches on users’ acceptance show that the perceived safety and trust are among the most relevant factors affecting Automated Vehicles (AVs) acceptance (Xu et al., 2018, Zhang et al., 2019), and that the experience with AVs can increase trust. Different large-scale surveys highlighted that people valued very highly being able to take over control of an AV (European Commission, 2020, Nordhoff et al., 2018), and that this willingness to take over control of an AV depends on specific socio-demographic characteristics (Grosso et al., 2021b). The respondents desire a level of control over the vehicle behavior, arguably because of the lack of trust. Similarly, passengers experiencing a ride with an automated shuttle would often use an emergency button inside the vehicle, just to see how the vehicle would react (Nordhoff et al., 2020). Therefore, safety and security should be clearly and transparently ensured, required by regulation (Fagnant and Kockelman, 2015) and supported by insurance schemes (Baumann et al., 2019, Grosso et al., 2021a).

Policies on AVs can help accelerate the development, reduce control uncertainties, and smoothen the migration from conventional to AV traffic (Li et al., 2019). Some of the first relevant regulations for AV targeted on-road testing, with different countries having very distinctive approaches (Lee and Hess, 2020). Moreover, on-road testing alone may not be sufficient to prove the increased safety of ADS. For human drivers, a fatal crash happens with a return period of 3.4 million hours of driving, which corresponds to a vehicle driving for 380 years straight (Shladover and Nowakowski, 2019). Karla and Paddock have evaluated that, for demonstrating that AVs are safer than human drivers, a fleet of 100 AVs should be tested for 24 h a day, for about 225 years (Kalra and Paddock, 2016). Changes in software or hardware could require the repetition of the test.

In this light, novel ways to evaluate and assure safety by setting proper requirements are required, and Traffic Conflict Techniques (TCTs) can be useful. TCTs have been developed to assess safety by identifying conflicts (Laureshyn et al., 2010), that are dangerous situations with much higher frequency than that of accidents. Moreover, the frequency of conflicts is correlated with the frequency of accidents for human drivers (Tarko, 2018). However, this correlation may change with the introduction of AVs, as different kinds of accidents may occur and the human driver behavior may change (Wang et al., 2021). One of the most widely used safety metrics of TCT is the Time-To-Collision (TTC) (Laureshyn et al., 2016, Mahmud et al., 2019, Wang and Stamatiadis, 2014). Using TTC, different traffic states can be identified in the famous NGSIM database (Kovvali et al., 2007), with the states shown to correspond to different levels of risk (Zhao et al., 2018). The potential risk for human drivers in dangerous car following situations can be assessed (Liu et al., 2019). TTC has been shown to act complementary to the calculation of minimum time headway, as the two values are suitable for different purposes (Vogel, 2003). Moreover, TTC together with time headway and final relative distance under braking, have been used to create Fuzzy rules, based on the Virginia 100-car database (Xiong et al., 2019). Different models have been developed to evaluate risks in situations that include lane-changing maneuvers, based on real data (Park et al., 2018, Xie et al., 2019).

TTC-based models have been used in safety evaluations for active safety of Advanced Driver Assistance Systems (ADAS) using experimental data, as in the work of Seiniger et al. (Seiniger et al., 2013). However, in contrast with ADAS systems which should intervene at the last moment in an emergency, AVs are responsible to avoid emergencies, when possible, by driving defensively. Those decisions are made on a tactical level, hence the term “Tactical Safety” has emerged (Schoener, 2020). This is an important difference in the responsibility and the performance evaluation of different levels of vehicle automation.

More elaborate metrics can consider estimating the stopping distance (Brunson et al., 2002, Doi et al., 1994), or even trajectory optimization techniques (Junietz et al., 2018). Additionally, model predictive safety metrics have been developed (Weng et al., 2020). The notion of safety force fields has also been introduced, which can be useful for pre-collision warning systems (Wang et al., 2016) and path planning (Rasekhipour et al., 2017, Wolf and Burdick, 2008). Safety field metrics can also carry information on the potential accident severity (Mullakkal-Babu et al., 2017). A safety force field algorithm has been suggested and verified through simulation by NVIDIA (NVIDIA, 2019). Moreover, the concept of a safety field has been used to model human like driving behavior in non-emergency conditions (Kolekar et al., 2020), or coupled with a behavior prediction model for the surrounding traffic, to overcome some disadvantages of traditional safety metrics (Wang et al., 2022). Such prediction schemes can be also based on naturalistic data, and be useful for the design or the test of an ADS (Feng et al., 2021), but for the design of regulation requirements, it may be desirable to avoid them. Safety relevant choices can be made not only on the tactical level but also on the strategic level, by planning a trip that avoids the routes with locations of high-risk (Ryan et al., 2020).

All the aforementioned papers provide significant contributions for accident analysis or controller design but cannot be directly used in the development of regulation requirements, which need additional information. A performance requirement, indeed, provides the set of conditions in which a collision shall be avoided or mitigated by the ADS. Therefore, the definition of sets of possible events is necessary. Those could be derived by observations in real data, by extracting correlations (Thal et al., 2020), or sample high-risk situations (Akagi et al., 2019). For the upcoming Level 3 ALKS, the riskiest cases will arguably be receiving a reckless cut-in by a slower vehicle, and a formulation to classify preventable and unpreventable cases is already in the text of the regulation [paragraph 5.2.5.2,(UNECE, 2021)]. In the same regulation, in Appendix 3, there is a “human driver” model, defined for cut-in, cut-out, and car-following scenarios. The model, which will be described in detail in the following sections, is proposed to simulate the behavior of a Competent and Careful (CC) human driver. The rationale is that the ALKS should guarantee at least the same level of safety of a careful and competent human driver, and therefore that any accident avoidable by that type of human driver, shall be also avoided by the ADS. This first CC human driver model is a major step towards a viable regulation framework, establishing a way to form performance requirements using simulation and assuming an intuitive driving style (VMAD, 2019a). Moreover, the developers of the CC human driver model provided estimations of the value of its parameters on the basis of extensive empirical observations (VMAD, 2019b).

Another model that could be used in safety regulation is the Responsibility Sensitive Safety (RSS) model, developed by Intel/Mobileye (Shalev-Shwartz et al., 2017). The model provides rules that could be superimposed to any AV control strategy, to make sure that the AV would not be responsible to cause an accident. The longitudinal safety control part of the model is similar to a formally verified control strategy (Loos et al., 2011). Even though RSS represents an operational requirement, it may be used to define the sets of preventable and unpreventable conditions through simulation, providing a performance requirement similarly as in the CC human driver model. RSS includes some parameters and an effort has been carried out to calibrate and evaluate the model based on two hundred cut-in events from the Shanghai Naturalistic Driving Study data (Liu et al., 2021). In the same work, it has been observed that human drivers would predict danger and adopt preventive strategies, which is not seen in the formulation of the CC driver model.

This type of performance requirement is suitable for the next developments in ADS regulation. Until the use cases considered by the regulation remain sufficiently simple and relatively easy to be described, it is indeed possible to introduce a set of conditions where collisions should be avoided in the riskiest traffic situations. However, “Tactical Safety” should be explicitly taken into account when using as performance reference the concept of a careful and competent human driver, though. Furthermore, apart from classifying whether a certain scenario would lead to a collision or not, the model should also be able to assess how challenging it is to avoid a collision, in order to offer a support tool in the choice of the scenario(s) to be tested. Therefore, such a model should classify preventable and unpreventable cases in a binary way, while also indicating a non-binary value of risk or severity. In this light, a novel model is presented here, based on Fuzzy Surrogate Safety Metrics (SSMs). The Fuzzy SSMs have been recently developed and verified using real data (Mattas et al., 2020, Mattas et al., 2019). The model can be used to set performance requirements, similarly to the CC human driver model in the existing regulation. At the same time, on the basis of the fuzzy membership value, it also embodies a direct way to define a level of difficulty in avoiding the collision.

## 3 Models

Four models are presented and compared in the paper, the two that are already part of Regulation 157, the RSS model proposed by Intel/Mobileye, and the Fuzzy Safety Model (FSM), originally proposed in this work. The model described in paragraph 5.2.5.2. Regulation 157 is referred to as Reg157 model, while the model in Appendix 3 of the same regulation will be referred hereinafter as the CC human driver model.

### 3.1 Reg157 model

The model, described in paragraph 5.2.5.2. refers to collision avoidance when another vehicle is cutting-in. If the cutting-in vehicle has a lower speed than the ALKS vehicle, and the lateral movement of the cutting-in vehicle has been visible for at least 0.72 sec, a collision should be avoided when the following equation (1) holds:

(1) $$\mathrm{TT}C>vrel/\left(2\times 6m/s^{2}\right)+0.35s$$

where TTC is the time to collision between the two vehicles and vrel is their relative velocity. Equation (1) is evaluated when the front wheel of the cutting-in vehicle is closer than 0.3 m to the outside edge of the visible lane markings. The assumption is that the maximum deceleration should be at least 6 m/s^2^. Moreover, the perception time, together with the time needed to achieve the deceleration of 6 m/s^2^ is equal to 0.35 s for a vehicle with high-level automated functionalities.

A simulation model is developed, based on this behavior so that the Reg157 for cut-in safety can be directly compared to the rest. In each simulation step, the ego vehicle is evaluating the lateral distance to the cutting-in vehicle. If the distance is unsafe, so the cutting-in vehicle’s edge is 0.3 m inside the ego vehicle’s lane, the TTC is calculated on the longitudinal dimension. If the TTC is large enough, and equation (1) is satisfied, an abrupt deceleration is not needed, as the situation is safe. On the other hand, if the TTC is small, the ego vehicle decelerates, with 6 m/s^2^, 0.35 s after the danger is identified. During this reaction time, the ego vehicle speed is assumed to be constant.

### 3.2 CC human driver model

In Appendix 3 of Regulation 157, a simulation model is presented, which assumes to mimic the behavior of a Competent and Careful human driver for three different critical situations: a) a vehicle cutting-in; b) a deceleration scenario in which the vehicle preceding the ego vehicle suddenly decelerates; c) a cut-out scenario in which the vehicle preceding the ego vehicle suddenly exits the lane, to reveal an obstacle.

For the cut-in scenario, the wandering distance of a vehicle inside its lane is defined to be 0.375 m, estimated from real data (JAMA, 2020). When a vehicle exits the wandering zone, it is assumed that it is about to initiate a lane change. An additional perception distance of 0.72 m is assumed. Once the vehicle in the adjacent lane is further than 0.72 m from the wandering zone, the CC human driver perceives the possible risk. The reaction time of the driver, estimated to be 0.75 s, starts at this point. During this time, the human driver moves his/her foot from the acceleration pedal. A small deceleration of 0.4 m/s^2^ is assumed for this period. After that, the maximum deceleration is estimated to be 0.774 g (7.59 m/s^2^), and it takes 0.6 s to be realized. During those 0.6 s, the deceleration is increasing linearly, in absolute value, corresponding to a maximum absolute jerk value of 12.65 m/s^3^. An additional constrain is that the CC driver would not start the emergency deceleration if the TTC is more than 2 s, as the situation is not considered to be an emergency, and a hard deceleration would not be necessary.

The formulation of the cut-out scenario is similar. The CC human driver perceives the cut-out when the preceding vehicle exceeds the lateral wandering distance. The deceleration dynamics are the same. For the simulation of the deceleration scenarios, the perception and reaction time start when the preceding vehicle starts decelerating. For both those cases, the ego vehicle maintains a 2 s time headway with the preceding vehicle, before the preceding vehicle changes its trajectory.

### 3.3 RSS model

The Responsibility Sensitive Safety model, proposed by Intel/Mobileye is a complete framework that can be used for a wide range of traffic situations. The main idea behind the framework is that the AV could not ensure absolute safety, but certain rules could ensure that the AV would not cause an accident. RSS is a rule-based system, to be combined with any controller. However, in this work, an RSS abiding driver model is simulated, to classify preventable and unpreventable scenarios, using simulation.

Only the paragraphs relevant to multi-lane freeways are considered in the present work. Two safety distances are introduced, the longitudinal and lateral safety distance, as shown in equations (2)–(3).

(2) $$d_{\mathrm{lon}}=u_{r}\rho +\frac{1}{2}a_{\max,accel}\rho^{2}+\frac{{\left(u_{r}+\rho a_{\max,accel}\right)}^{2}}{2a_{\min,brake}}-\frac{u_{f}^{2}}{2a_{\max,brake}}$$

(3) $$d_{\mathrm{lat}}=\mu +\frac{{2u}_{1}+\rho {a^{\mathrm{lateral}}}_{\max,accel}}{2}\rho +\frac{{\left(u_{1}+\rho {a^{\mathrm{lateral}}}_{\max,accel}\right)}^{2}}{2{a^{\mathrm{lateral}}}_{\min,brake,correct}}-\frac{{2u}_{2}+\rho {a^{\mathrm{lateral}}}_{\max,accel}}{2}\rho +\frac{{\left(u_{2}+\rho {a^{\mathrm{lateral}}}_{\max,accel}\right)}^{2}}{2{a^{\mathrm{lateral}}}_{\min,brake,correct}}$$

where $d_{\mathrm{lon}}$ is the minimum safe longitudinal distance, $u_{r}$ and $u_{f}$ are the speeds of the ego vehicle (rear), and the preceding vehicle (front) respectively, ρ is the reaction time of the ego vehicle, $a_{\max,accel}$ is the maximum acceleration of the ego vehicle, $a_{\min,brake}$ is an estimation of the ego vehicle’s maximum deceleration, and $a_{\max,brake}$ and estimation of the preceding vehicle’s maximum deceleration, with the constrain that $a_{\min,brake}$ is always smaller in absolute value than $a_{\max,brake}$, $d_{\mathrm{lat}}$ is the minimum safe lateral distance, μ a lateral safety distance margin, $u_{1}$ and $u_{2}$ the lateral speeds of the ego and preceding vehicle, ${a^{\mathrm{lateral}}}_{\max,accel}$ and ${a^{\mathrm{lateral}}}_{\min,brake,correct}$ maximum and minimum absolute values of lateral acceleration.

If at least one of the safety distances is respected, the situation is safe. A reaction is required by the ego vehicle only in case both safety distances are smaller than the relevant safe distance. The proper reaction depends on which was the latest safety distance that was respected. If it was the longitudinal distance, the proper reaction is a deceleration with at least $a_{\min,brake}$ after a time equal to ρ. On the other hand, if it was the lateral distance, a possibility for a dangerous cut-in is recognized and the proper reaction is to decelerate on the lateral direction if the ego vehicle has any lateral speed towards the other vehicle. If by doing this the ego vehicle does not exit the unsafe area, even when the lateral speed of the ego vehicle is 0, then a longitudinal deceleration of at least $a_{\min,brake}$ is required. In the simulations carried out, the deceleration increases linearly, exactly as in the CC human driver model.

For the simulation of the cut-out and the deceleration scenarios, the initial distance between the ego vehicle and the preceding vehicle is within the control of the AV driving strategy. For the RSS simulation model, the time headway should not be equal to the minimum safe longitudinal distance, as this would mean that in the process of achieving and maintaining the steady-state headway, the ego vehicle could enter the unsafe distance. The same would happen in case of a deceleration of the preceding vehicle, even if it is mild. This would be against the RSS rules. Hence, the equilibrium distance is calculated such that the ego vehicle will not enter the unsafe distance after a time equal to ρ, if the preceding vehicle decelerates with $a_{\max,brake}$ for this time, as shown in equation (4).

(4) $$d_{\mathrm{time}-headway}=u_{r}\rho +\frac{1}{2}a_{\max,accel}\rho^{2}+\frac{{\left(u_{r}+\rho a_{\max,accel}\right)}^{2}}{2a_{\min,brake}}-\frac{{\left(u_{f}-\rho a_{\max,brake}\right)}^{2}}{2a_{\max,brake}}+\frac{1}{2}a_{\max,brake}\rho^{2}\;$$

### 3.4 The fuzzy safety model

The Fuzzy Safety Model (FSM) is based on fuzzy surrogate safety metrics for rear-end collisions. The simulation model consists of three steps. First, the lateral safety distance is checked, and if there is no potential risk identified, no reaction is required by the ego vehicle. Otherwise, the process continues by checking the longitudinal distance. This first step is necessary only for the cut-in scenarios. For cut-out and deceleration scenarios, it is skipped, as the vehicles are in the same lane and the lateral distance is always potentially dangerous, so the process starts from checking the longitudinal distance. If a risk is identified according to the longitudinal distance check, the proper reaction is calculated, in the term of a deceleration value to be achieved. The check is carried out in every simulation step.

The lateral safety check uses three criteria. If all criteria are true, a potential risk is identified. The three criteria are:

• The cutting-in vehicle is downstream.
• The cutting-in vehicle has lateral speed towards the ego vehicle.
• The following is true:
  o If the longitudinal velocity of the ego vehicle is greater than the longitudinal velocity of the cutting-in vehicle, equation (5):

(5) $$\frac{\mathrm{dis}t_{\mathrm{lat}}}{u_{\mathrm{cut}-in,lat}}<\frac{\mathrm{dis}t_{\mathrm{lon}}+length_{\mathrm{ego}}+length_{\mathrm{cut}-in}}{u_{\mathrm{ego},lon}-u_{\mathrm{cut}-in,lon}}+s_{1}$$

o If the longitudinal velocity of the ego vehicle is lower than the longitudinal velocity of the cutting-in vehicle, equation (6):

(6) $$\frac{\mathrm{dis}t_{\mathrm{lat}}}{u_{\mathrm{cut}-in,lat}}<\frac{x_{\mathrm{ego},front}-x_{\mathrm{cut}-in,back}}{u_{\mathrm{cut}-in,lon}-u_{\mathrm{ego},lon}}+s_{1}$$

where $\mathrm{dis}t_{\mathrm{lat}}$ is the lateral distance between the two vehicles, $\mathrm{dis}t_{\mathrm{lon}}$ is the longitudinal distance between the two vehicles, $\mathrm{lengt}h_{\mathrm{ego}}$ and $\mathrm{lengt}h_{\mathrm{cut}-in}$ the length of the ego vehicle and the cutting-in vehicle respectively, $s_{1}$ is a time safety margin of 0.1 s, $u_{\mathrm{cut}-in,lat}$ is the lateral velocity of the cutting-in vehicle, $u_{\mathrm{ego},lon}$ and $u_{\mathrm{cut}-in,lon}$ the longitudinal velocity of the ego vehicle and the cutting-in vehicle respectively. For the second case, $x_{\mathrm{ego},front}$ is the longitudinal position of the center of the front bumper of the ego vehicle and $x_{\mathrm{cut}-in,back}$ is the longitudinal position of the center of the back bumper of the cutting-in vehicle.

The lateral safety check evaluates the lateral movement of the cutting-in vehicle. Assuming no reaction, the position of the cutting vehicle in the trajectory of the ego vehicle is estimated. For small lateral speeds, it is possible that this position would be behind the ego-vehicle, and there would be no risk of an accident. Otherwise, there is danger of the cutting-in vehicle crashing to the side of the ego-vehicle or getting in front of it in an unsafe distance, such that the ego-vehicle would not be able to decelerate enough to avoid an accident. There is a safety margin of 0.1 s that is further explained in the results section.

Two fuzzy surrogate safety metrics are evaluated for the longitudinal safety check, the Proactive Fuzzy surrogate Safety metric (PFS) and the Critical Fuzzy surrogate Safety metric (CFS) (Mattas et al., 2020). If both metrics take the value 0, the situation is safe, and no reaction is required. If any of the two takes a non-zero value, the condition is not safe, and a reaction is required.

The value of PFS is calculated according to equation (7):

(7) $$PFS\left(dist_{\mathrm{lon}}\right)=\;\left\{\begin{array}{cc} 1, & if\;0<dist_{\mathrm{lon}}-d_{1}<d_{\mathrm{unsafe}}\\ 0, & if\;dist_{\mathrm{lon}}-d_{1}>d_{\mathrm{safe}}\\ \frac{\mathrm{dis}t_{\mathrm{lon}}-d_{\mathrm{safe}}-d_{1}}{d_{\mathrm{unsafe}}-d_{\mathrm{safe}}}, & if\;d_{\mathrm{unsafe}}<dist_{\mathrm{lon}}-d_{1}<d_{\mathrm{safe}} \end{array}\right)$$

where $\mathrm{dis}t_{\mathrm{lon}}$ is the longitudinal distance between the two vehicles, $d_{1}$ is a safety margin of 2 m, and $d_{\mathrm{safe}}$, $d_{\mathrm{unsafe}}$ are calculated according to equations 8–9:

(8) $$d_{\mathrm{safe}}=u_{\mathrm{ego},lon}\tau +\frac{u_{\mathrm{ego},lon}^{2}}{2b_{\mathrm{ego},comf}}-\frac{u_{\mathrm{cut}-in,lon}^{2}}{2b_{\mathrm{cut}-in,max}}+d_{1}$$

(9) $$d_{\mathrm{unsafe}}=u_{\mathrm{ego},lon}\tau +\frac{u_{\mathrm{ego},lon}^{2}}{2b_{\mathrm{ego},max}}-\frac{u_{\mathrm{cut}-in,lon}^{2}}{2b_{\mathrm{cut}-in,max}}$$

with $u_{\mathrm{ego},lon}$ the velocity of the ego vehicle, $u_{\mathrm{cut}-in,lon}$ the velocity of the cutting-in vehicle, τ the reaction time of the ego vehicle, $b_{\mathrm{ego},comf}$ the comfortable deceleration of the ego vehicle,$\;b_{\mathrm{ego},max}$ the maximum deceleration of the ego vehicle, $b_{\mathrm{cut}-in,max}$ the maximum deceleration of the cutting-in vehicle, $d_{1}$ a safety distance margin of 2 m.

The value of CFS is calculated only when the ego vehicle velocity is higher than the preceding vehicle, according to equation (10):

(10) $$CFS\left(dist_{\mathrm{lon}}\right)=\;\left\{\begin{array}{cc} 1, & if\;0<dist_{\mathrm{lon}}<d_{\mathrm{unsafe}}\\ 0, & if\;dist_{\mathrm{lon}}>d_{\mathrm{safe}}\\ \frac{\mathrm{dis}t_{\mathrm{lon}}-d_{\mathrm{safe}}}{d_{\mathrm{unsafe}}-d_{\mathrm{safe}}}, & if\;d_{\mathrm{unsafe}}<dist_{\mathrm{lon}}<d_{\mathrm{safe}} \end{array}\right)$$

where $\mathrm{dis}t_{\mathrm{lon}}$ is the longitudinal distance between the two vehicles, and $d_{\mathrm{safe}}$, $d_{\mathrm{unsafe}}$ are calculated according to equations 11–15:

(11) $$d_{\mathrm{safe}}=\;\left\{\begin{array}{c} \frac{{\left(u_{\mathrm{ego},lon}-\;u_{\mathrm{cut}-in,lon}\right)}^{2}}{-2a_{\mathrm{ego}}^{\prime }},ifu_{\mathrm{ego},lon,NEXT}\leq u_{\mathrm{cut}-in,lon}\\ d_{\mathrm{new}}+\frac{{\left(u_{\mathrm{ego},lon,NEXT}-u_{\mathrm{cut}-in,lon}\;\right)}^{2}}{2b_{\mathrm{ego},comf}},ifu_{\mathrm{ego},lon,NEXT}>u_{\mathrm{cut}-in,lon} \end{array}\right)$$

(12) $$d_{\mathrm{unsafe}}=\;\left\{\begin{array}{c} \frac{{\left(u_{\mathrm{ego},lon}-\;u_{\mathrm{cut}-in,lon}\right)}^{2}}{-2a_{\mathrm{ego}}^{\prime }},ifu_{\mathrm{ego},lon,NEXT}\leq u_{\mathrm{cut}-in,lon}\\ d_{\mathrm{new}}+\frac{{\left(u_{\mathrm{ego},lon,NEXT}-u_{\mathrm{cut}-in,lon}\;\right)}^{2}}{2b_{\mathrm{ego},max}},ifu_{\mathrm{ego},lon,NEXT}>u_{\mathrm{cut}-in,lon} \end{array}\right)$$

(13) $$a_{\mathrm{ego}}^{\prime }=max\left(a_{\mathrm{ego}},{-b}_{\mathrm{ego},comf}\right)$$

(14) $$u_{\mathrm{ego},lon,NEXT}=u_{\mathrm{ego},lon}+a_{\mathrm{ego}}^{\prime }\tau$$

(15) $$d_{\mathrm{new}}=\left(\frac{{(u}_{\mathrm{ego},lon}+u_{\mathrm{ego},lon,NEXT})}{2}-u_{\mathrm{cut}-in,lon}\right)\tau$$

with $u_{\mathrm{ego},lon}$ the velocity of the ego vehicle, $u_{\mathrm{ego},lon,NEXT}$ the expected velocity of the ego vehicle after the reaction time assuming constant acceleration, $a_{\mathrm{ego}}$ the current acceleration of the ego vehicle, ${a\prime }_{\mathrm{ego}}$ a modified acceleration of the ego vehicle that cannot be harder than $b_{\mathrm{ego},comf}$, $d_{\mathrm{new}}$ is the expected change in the distance between the two vehicles after the response time, $u_{\mathrm{cut}-in,lon}$ the velocity of the cutting-in vehicle, τ the reaction time of the ego vehicle, $b_{\mathrm{ego},comf}$ the comfortable deceleration of the ego,$\;b_{\mathrm{ego},max}$ the maximum deceleration of the ego vehicle.

The deceleration intensity is relative to the values of PFS and CFS. The reaction is that of a simplified rule-based Fuzzy Inference System, as in equation (16).

(16) $$b_{\mathrm{reaction}}=\left\{\begin{array}{cc} CFS\left(b_{\mathrm{ego},max}-b_{\mathrm{ego},comf}\right)+b_{\mathrm{ego},comf}, & if\;CFS>0\\ PFSb_{\mathrm{ego},comf}, & if\;CFS=0 \end{array}\right)$$

where CFS and PFS are the values calculated in equations (10), (7) respectively. The reaction starts after a time equal to τ, and the deceleration value increases linearly, exactly as in the CC human driver model. A significant difference between this model to the other three is that the simulated driver may react using a calm deceleration, in anticipation of an emergency maneuver. For all the other models, the decelerations required by the control strategy are either 0 or the maximum.

## 4 Assessment methodology

### 4.1 Simulation framework

A simulation framework is developed in Python, to test the four different models. The three types of scenarios, as previously described, are a) cut-in, b) cut-out, and c) deceleration of the preceding vehicle. The trajectories of the other vehicles are pre-determined to fit the scenario and vary depending on different initial conditions. The simulated vehicle keeps constant longitudinal velocity and 0 lateral velocity unless required to decelerate by the model used each time. All trajectories are stored to investigate the level of challenge in each situation. The same rules apply to the simulation of the real-world trajectories for the validation. The parameter values used for the Reg157 model, and the CC human driver model are as presented in the Regulation 157. For the RSS and FSM simulations, the parameters are presented in Table 1 and Table 2, respectively. They are corresponding to the CC human driver model where is fitting. However, the parameter values can be subject to discussion and change. In the present paper, the attributes and qualitative results of each model are compared.

**Table 1:** Value of the parameters used in the simulations involving the RSS model.

Parameter	RSS
ρ	0.75 s
$a_{\max,accel}$	3 m/s^2^
$a_{\min,brake}$	6 m/s^2^
$a_{\max,brake}$	6 m/s^2^
μ	0.3 m
${a^{\mathrm{lateral}}}_{\max,accel}$	1 m/s^2^
${a^{\mathrm{lateral}}}_{\min,brake,correct}$	1 m/s^2^
Max abs deceleration	0.774 g
Max abs jerk	12.65 m/s^3^

**Table 2:** Value of the parameters used in the simulations involving the FSM.

Parameter	FSM
τ	0.75 s
$b_{\mathrm{ego},comf}$	3 m/s^2^
$b_{\mathrm{ego},max}$	6 m/s^2^
$b_{\mathrm{cut}-in,max}$	7 m/s^2^
Max abs deceleration	6 m/s^2^
Max abs jerk	12.65 m/s^3^

### 4.2 Replication of regulation example cases

For all three types of scenarios, the examples presented in Appendix 3 of the regulation (UNECE, 2021) are recreated, with additional simulations for higher speed, up to 130 km/h to widen the scope of the assessment to the whole motorway driving. The simulation framework regarding the regulatory application is openly available^2^. In the cut-in scenarios, the simulations are extended backward in time, so the cutting-in vehicle starts with 0 lateral speed and accelerates with a lateral acceleration of 1.5 m/s^2^. The distances are calculated so when the lateral distance is equal to the scenario setting (1.6 m for all tests as in Appendix 3), the lateral velocity and longitudinal distance of the vehicles has just reached the indicated value. This is crucial for the RSS and FSM, which could react in an anticipatory fashion. This would probably fit the way the experiments would be carried out on a test track. For all models except the Reg157, the jerk is bounded, so the deceleration of the ego vehicle is decreasing linearly when a risk is identified.

### 4.3 Validation using real data

Throughout the paper, real data are used for comparison purposes, for fixing parameter values, and, most importantly, for the comparison of the models’ efficiency. The data are from the highD database (Krajewski et al., 2018). The human driver trajectories in the database are collected on German highways, at six different locations near Cologne, using unmanned aerial vehicles. The locations vary by the speed limits and the number of lanes. The typical position error of the detected vehicles is 10 cm.

The main limitation of this work is the small sample size of data. Fatal traffic accidents are rare (Kalra and Paddock, 2016) and a vastly larger dataset should be used. In fact, there are no accidents reported in the highD dataset. Moreover, as any human driver drives differently under different conditions, a perfect deterministic model of a competent and careful human driver cannot exist. Due to those limitations, a parameter calibration is not carried out. The validation part aims to investigate the different mechanics of each model and to show why and how the proposed model is better suitable to describe the reaction of a competent and careful human driver.

The different models are not used to accurately replicate the behavior of a human driver, which is a challenging task. Instead, they are used to classify if a certain scenario should be considered preventable (i.e. not leading to a collision) or unpreventable (i.e. leading to a collision). Therefore, for the validation of the models, the way the human driver reacted to a situation does not necessarily need to be exactly replicated. On the contrary, what is important is that the scenarios that are preventable for a human driver in real life are also classified as preventable by the models. Furthermore, the models are also checked on cases in which there was no cut-in but two vehicles were driving closely in adjacent lanes, to investigate whether the models are too conservative and therefore also not suitable to be used as classifiers (as they would generate too many false-positive cases).

To examine the capacity of the models to correctly classify preventable cases, the most hazardous cut-in cases have been extracted from the highD dataset. The models should classify the cases as preventable, as the human drivers were able to avoid an accident and as there is no accident recorded in the highD dataset. Some assumptions are required to obtain the worst cases:

• The vehicle receiving the lane change needs to be classified as a car. Vehicles classified by the developers of the dataset as “truck” would not be used as the ego vehicle.
• The ego vehicle should not have changed lanes during the recording. Since every vehicle is recorded for a few seconds, a lane change by the vehicle receiving the cut-in might mean that this was not a “pure” cut-in maneuverer. Maybe the ego vehicle changed lanes to avoid a dangerous situation, and the models are not capable of replicating this movement. Therefore, any vehicle eligible to be considered as a vehicle receiving a cut-in must have been completely inside the lane markings for the whole recording.
• The cutting-in vehicle should start from another lane and end with at least one wheel in the ego vehicle’s lane. Moreover, it should not move to another lane during the recording. Those cases may correspond to a double lane change and not to a cut-in.
• The trajectories are obtained only for the period that there are recordings for both vehicles. Moreover, the time instance in which the cutting-in vehicle first touched the lane markings is identified. The parts of the trajectories referring to a timestamp that is more than 5 s earlier than this time instance are discarded as the behavior during this is irrelevant to the maneuver.
• The TTC is calculated for each instance of the 25 Hz recordings. Only the cases in which the TTC achieved a value of 5 s or less are obtained for the validation. This large TTC threshold was chosen to include cases where the driver’s proactive behavior prevented a more severe conflict.

Regarding the cases where no reaction would be necessary as the “other” vehicle did not perform a lane change, pairs of vehicles traveling in adjacent lanes are identified. Again, several assumptions are used:

• Both vehicles have to be always inside the markings of their respective lanes. If not, there are cases where one of the vehicles drifts in the other lane, without completing the lane change during the observed period, and the vehicle upstream would need to decelerate or move laterally to avoid a collision.
• The time for which there are data for both vehicles should be larger than 3 s, otherwise the maneuverer is too short to be used as an example.
• One of the two vehicles should always be in front of the other one, looking at their rightmost position from the highD data. Overtaking maneuvers are not relevant to the current models’ focus.

## 5 Results

In this section, the results of the simulated scenarios are presented. The 4 different reaction models are designed to estimate through simulation if a scenario is preventable or unpreventable. The Reg157 model is used only for the cut-in scenarios. Finally, the efficiency of the models regarding the cut-in scenarios is investigated using real trajectory data.

### 5.1 Cut-in scenarios

The simulations reconstruct the results presented in the regulation Appendix 3. The lateral distance between the two vehicles is 1.6 m. For the Reg157 and the CC human driver model, the position of the lane markings affects the time of reaction of the ego vehicle. For the simulation experiments presented, the lane markings are assumed to be exactly in the middle of the distance between the two vehicles. The rest of the parameters are presented in Table 3 for the low-speed scenarios and Table 4 for the high-speed scenarios.

**Table 3:** Parameter values for low-speed simulation experiments of cut-in.

	Minimum value	Maximum value	Step
Longitudinal distance	1 m	60 m	1 m
Cutting-in vehicle lateral velocity	0.1 m/s	1.8 m/s	0.1 m/s
Ego vehicle velocity	10 km/h	60 km/h	10 km/h
Cutting-in vehicle velocity	10 km/h	50 km/h	10 km/h

**Table 4:** Parameter values for high-speed simulation experiments of cut-in.

	Minimum value	Maximum value	Step
Longitudinal distance	1 m	120 m	2 m
Cutting-in vehicle lateral velocity	0.1 m/s	1.8 m/s	0.1 m/s
Ego vehicle velocity	70 km/h	130 km/h	20 km/h
Cutting-in vehicle velocity	10 km/h	100 km/h	30 km/h

**Fig. 1:**
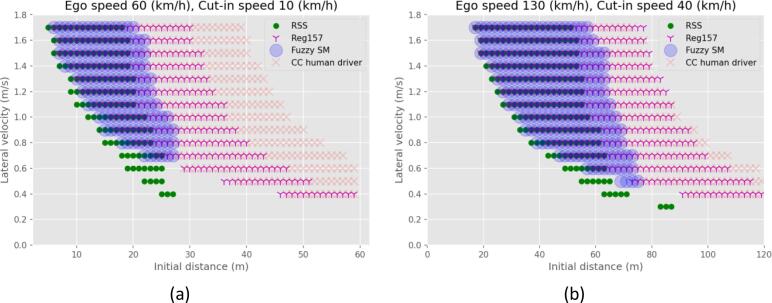
Unpreventable cases for all 4 models for the (a) ego vehicle running with 60 km/h and the cutting-in vehicle running with 10 km/h longitudinal speed, (b) ego vehicle running with 130 km/h, and the cutting-in vehicle running with 40 km/h longitudinal speed.

The most challenging combination of velocities for the low-speed scenarios is the cutting-in vehicle running with 10 km/h longitudinal velocity and the ego vehicle 60 km/h. The results regarding the preventability of each scenario for this combination, are presented in Fig. 1a, and an example for the high-speed scenarios with the cutting-in vehicle running with 40 km/h longitudinal velocity and the ego vehicle 130 km/h in Fig. 1b. The green dots represent the cases that have been judged to be unpreventable by the RSS model, the magenta “Y” markers regard the Reg157 unpreventable cases, the red “X” markers the CC human driver, and the blue transparent large dots represent the proposed FSM, for all combinations of the lateral velocity of the cutting-in vehicle and the initial longitudinal distance.

A first observation is that the unpreventable cases defined by the Reg157 and CC human driver models are similar. There is some difference, because of the different assumptions of the reaction time, maximum deceleration, and the different assumptions on when a vehicle initiates the reaction. The formulation of both models is based on calculating the TTC. Thus, the simulated driver reacts to emergencies. On the other hand, both the RSS model and the FSM model assume anticipative behavior. The velocity and stopping distance of both vehicles is used to foresee upcoming emergencies and react proactively. Hence, both the RSS and FSM avoid accidents when the initial distance is large and the cutting-in lateral velocity is small. While the model parameters are in essence assumptions, their values would not change this observation qualitatively. The current formulation of the CC human driver model and the Reg157 model is not capable of anticipating emergencies.

**Fig. 2:**
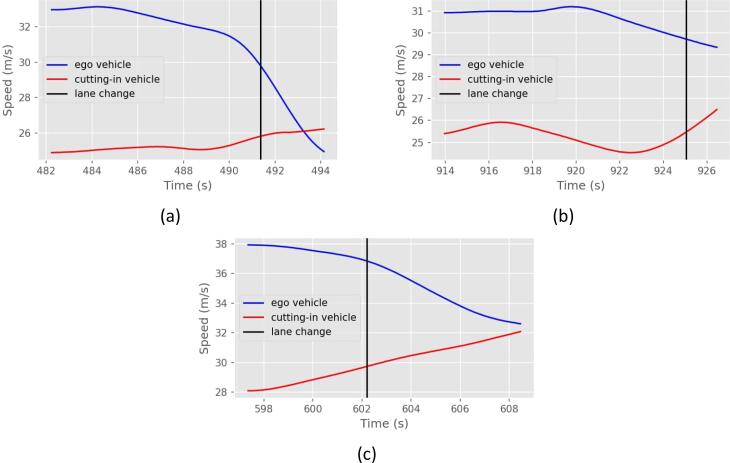
Three cases observed in the highD dataset, in which the human driver reacts in anticipation of a cut-in, using a soft deceleration.

The main argument for the use of a model that is capable of recreating anticipatory behavior is that human drivers are also capable of such behavior. This is confirmed by investigating lane changes in the highD data set. Three examples are presented in Fig. 2, with the red line denoting the longitudinal velocity of the cutting-in vehicle and the blue line the velocity of the vehicle receiving the cut-in. The time in which the closer side of the cutting-in vehicle reached the lane marking is denoted by the black, vertical line. In all three cases, the vehicle that received the cut-in was able to anticipate the maneuver and started decelerating a few seconds earlier. Since the data refer to human drivers, it can be assumed that their reaction time is non-negligible, and the situation has been predicted even earlier than when the deceleration was realized. Thus, it may be argued that AVs would be expected to have the same capabilities and avoid accidents by anticipation if a human driver would avoid it as well.

Two aspects separate RSS from the FSM. The right boundary of the unpreventable area for the RSS model is on the left of that of the FSM model. Hence, some cases that would be classified as preventable using the RSS, are unpreventable according to the FSM. The FSM uses calm deceleration when an emergency is predicted, while the RSS model can only react with a full deceleration. Hence, the RSS decelerates harder and can avoid an accident with slightly less space. This is directly affected by the assumed parameters of the models.

The second difference between the two modeled results is an area for small initial distance and low lateral velocity of the cutting-in vehicle, which is identified as unpreventable only by the RSS of all the models. From a visual inspection of the simulations, it becomes clear that for such cases, the longitudinal relative speed is large, and the approaching rate of the cutting-in vehicle is small. Hence, if the ego vehicle does not decelerate, it would be in front of the cutting-in vehicle when their trajectories intersect. However, the conservative behavior of the RSS requires the simulated vehicle to decelerate in anticipation, and a collision occurs.

These accidents are avoided with the FSM, because of the constrain in equation (5). When the inequality is not satisfied, the approaching rate of the cutting-in vehicle is too small compared to the relative speed, so the ego vehicle can avoid the accident by keeping a constant speed. In this area, small changes in the parameters can drastically change the result of the simulation. A small difference in the relative velocity of the vehicles could be the deciding factor between a very critical unpreventable situation and a situation that would be prevented without any corrective maneuvers. This discontinuity may lead to sensor and model uncertainties becoming important. Hence, the safety margin of 0.1 s is used in the equation (5), so these cases that are almost accidents are not considered preventable. Overall, the FSM is close to the intersection of the other three models showing a good compromise between the different approaches.

The results for other scenario parameters are qualitatively the same. The results for other cases are presented in this paper’s annex.

An advantage of using a simulation framework is that a complete trajectory is generated. Using the trajectory and relevant safety metrics, different scenarios can be classified for the level of challenge, and not just as being preventable or unpreventable. This can be useful for the regulation of ADS, as it should cover the testing procedures that a technical service should run to check for compliance. An idea discussed in the amendment of Regulation 157 is that the testing authority should be allowed to test any scenario and not only specific ones, to avoid designs fitted on specific test cases. Therefore, an evaluation of the challenge level for all preventable scenarios is important, to ensure that at least a minimum number of challenging scenarios will be tested.

**Fig. 3:**
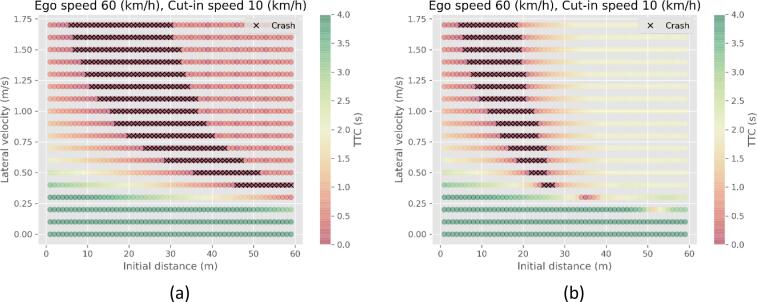
The minimum TTC value observed in the cut-in simulations for a) Reg157 model, b) RSS model.

Both the Reg157 model and CC human driver model use the TTC to identify emergencies and request a reaction. Therefore, the minimum TTC value observed would be very small, even for scenarios that are not very challenging. On the other hand, the RSS framework requests a very strong and early deceleration. An example of the results for the same speeds is presented in Fig. 3. The lower TTC values are denoted by the red coloring, and the higher, and thus safer, TTC values with green, for the Reg157 (a) and RSS model (b). For the Reg157 the TTC should be low to trigger a reaction so all cases of the vehicle using a deceleration to avoid an accident are colored red. On the other hand, the hard reaction of RSS shows cases that are close to the unpreventable front, to be much less challenging.

**Fig. 4:**
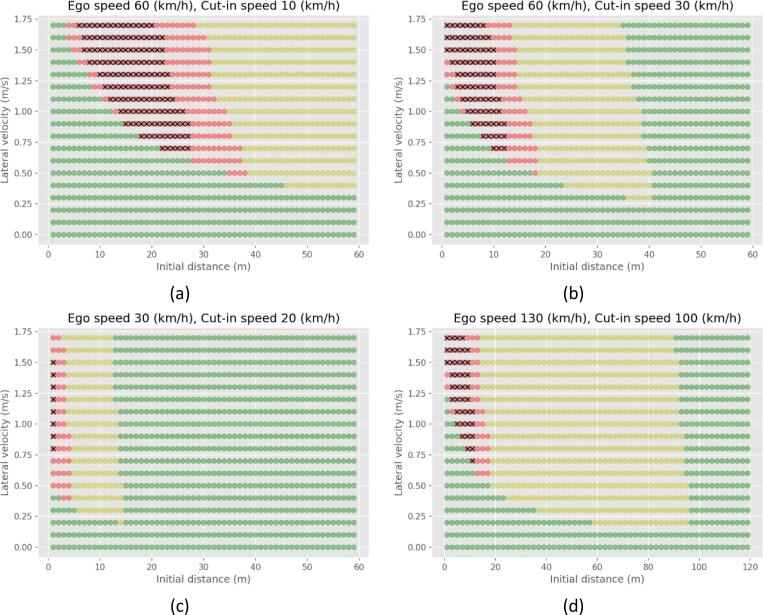
Classification of easy (green), medium (yellow), and difficult (red) scenarios according to the FSM.

The FSM is based on the PFS and CFS safety metrics. Thus, it naturally lends itself to classification in three different classes. When the CFS has achieved values close to 1, the scenario can be assumed to be very challenging. In the present example, the threshold of CFS to be higher than 0.9 is used. Otherwise, if large values of PFS have not been achieved, the scenario was rather easy to handle. The threshold used in this example is for PFS to achieve values lower than 0.85. Scenarios with values in-between can be classified as scenarios of medium challenge. Examples of the following classification are shown in Fig. 4. The combinations of velocities of the ego vehicle and the cut-in vehicle are 60–10, 60–30, 30–20, and 130–100 km/h in Fig. 4a,Fig. 4b, Fig. 4c, and Fig. 4d respectively. A distinct difference of the areas is presented, conditional to the velocity of the two vehicles. The results for the rest of the cases are presented in this paper’s annex.

### 5.2 Preceding vehicle sharp deceleration scenarios

Simulations of deceleration scenarios have been run for the CC human driver model, the RSS model, and the FSM. The scenarios have different initial conditions, as the steady-state distance required by each model is different. For the CC human driver, it is assumed to be 2 s time headway for any speed. The initial distance of the RSS model is according to equation 4. Finally, the initial distance of the FSM is conservatively assumed to be when PFS is 0.

Both the CC human driver model and the FSM did not find any unpreventable cases. On the other side, the RSS model identified a few when the speed is very high, and the deceleration of the preceding vehicle is much higher than the parameter assumed in this work. The RSS simulated driver with more conservative parameter values, especially regarding the maximum deceleration of the preceding vehicle, would also be able to avoid all accidents. The figures are presented in the annex of the current paper, along with the classification of different scenarios according to the level of challenge, when using the FSM. The results are intuitive as harder decelerations are classified as the most difficult cases.

### 5.3 Cut-out scenarios

The cut-out scenarios presented in the current regulation text consider only cases of the ego vehicle speed not exceeding 60 km/h. The variables are the lateral speed of the preceding vehicle and the distance in front of the preceding vehicle where a static obstacle is located. The ego vehicle and preceding vehicle have the same speed and the headway is assumed to be the steady-state one. The preceding vehicle starts a lateral movement to change lane, revealing a static vehicle (obstacle) in a certain distance in front of it. The ego vehicle may start reacting when the preceding vehicle has deviated more than 0.375 m, which is the wandering zone. The reaction will not be realized before a time equal to the reaction time of each model.

**Fig. 5:**
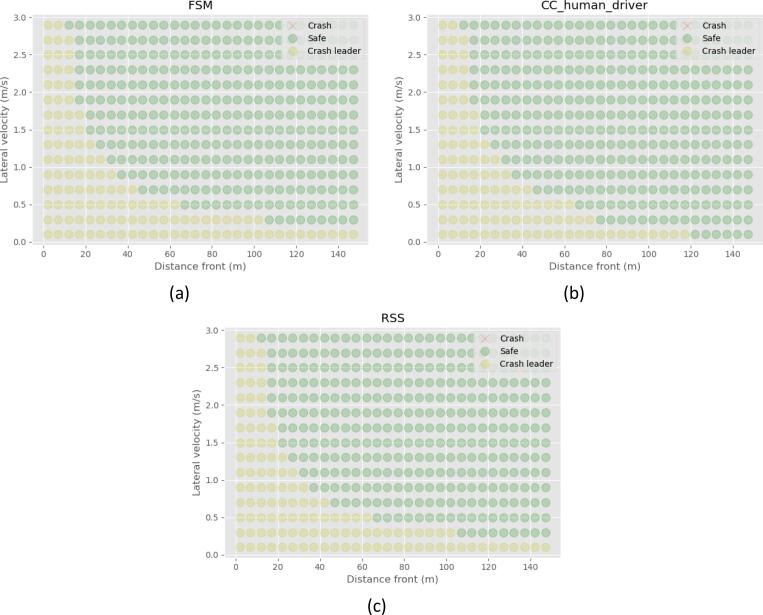
Results of cut-out scenarios with the ego vehicle speed equal to 60 km/h using a) FSM, b) CC human driver, c) RSS.

For such scenarios, no unpreventable cases are predicted in the regulation text. Moreover, the CC human driver is showing all cases to be avoidable. This is confirmed also by the FSM and RSS model, as shown in Fig. 5*a*, *b*, and *c* for the FSM, CC human driver, and RSS models, respectively. Green dots represent cases where the accident is prevented, and yellow dots represent cases of the preceding vehicle crashing into the static object. Crashes would be denoted by red ‘x’ markers, but there have been non in this case.

**Fig. 6:**
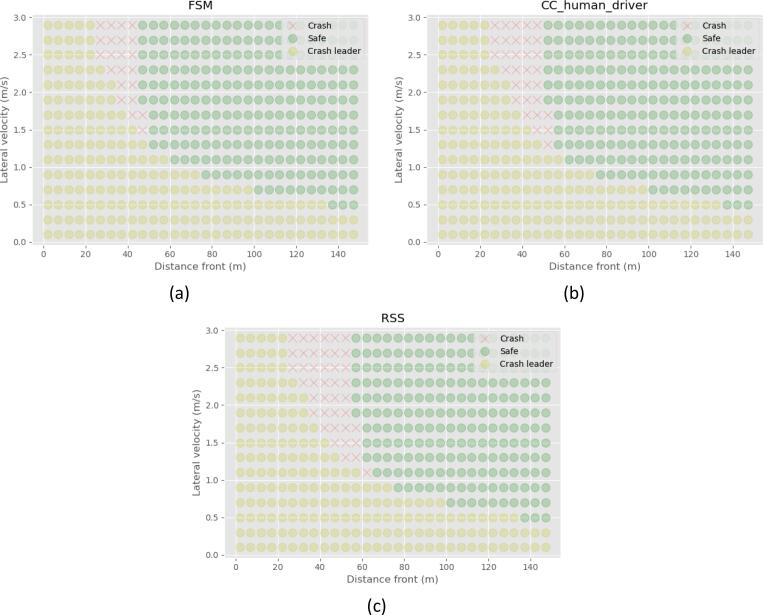
Results of cut-out scenarios with the ego vehicle speed equal to 130 km/h using a) FSM, b) CC human driver, c) RSS.

The regulation text does not suggest a model for the cut-out scenarios, assuming that all cases should be avoidable. For higher speeds though, the situation is more critical. In Fig. 6, the relevant results are presented for 110 km/h. For all three models, there are a few cases that would be regarded as unavoidable. For this case, in contrast with the cut-in case, tactical decision-making would not lead to less unpreventable scenarios. The risk is not known before the preceding vehicle exits the wandering zone, so there cannot be an anticipatory deceleration. Moreover, for all the unpreventable cases, the emergency deceleration was not enough, or the reaction time was not short enough. Therefore, all model formulations are equally capable in the classification of different cases for cut-out, and the reaction time and maximum deceleration parameters are crucial in the result. The results of the rest of the cases are presented in this paper’s annex.

Again, FSM has the advantage of classifying the different situations according to the level of challenge. In this specific case, a disadvantage of the simplistic formulation can affect the results of the classification, but not of the distinction between preventable and unpreventable. With a static obstacle upstream, the values of PFS and CFS are very similar. As a result, the CFS values increase, before the vehicle starts taking action to avoid the collision. Hence, the CFS value will be large for almost all of the simulated scenarios. This drawback could be eliminated by using a more complex control strategy, based on the Fuzzy SSMs. However, it may be counter-productive to use different model formulations for different cases or adopt more complicated models containing more parameters whose values need to be established. Hence, the situations will not be classified by the maximum value of CFS and PFS, but by the value of CFS and PFS at the time that the ego vehicle driver identifies the danger when the preceding vehicle is for the first instance out of the wandering zone. The thresholds for the classification of the different values are thus different. Cases of PFS and CFS equal to 0 are considered easy. Scenarios of medium challenge are those who have a CFS value that is less than 0.5. The cases of the CFS value being larger than 0.5 are assumed to be the most challenging situations.

**Fig. 7:**
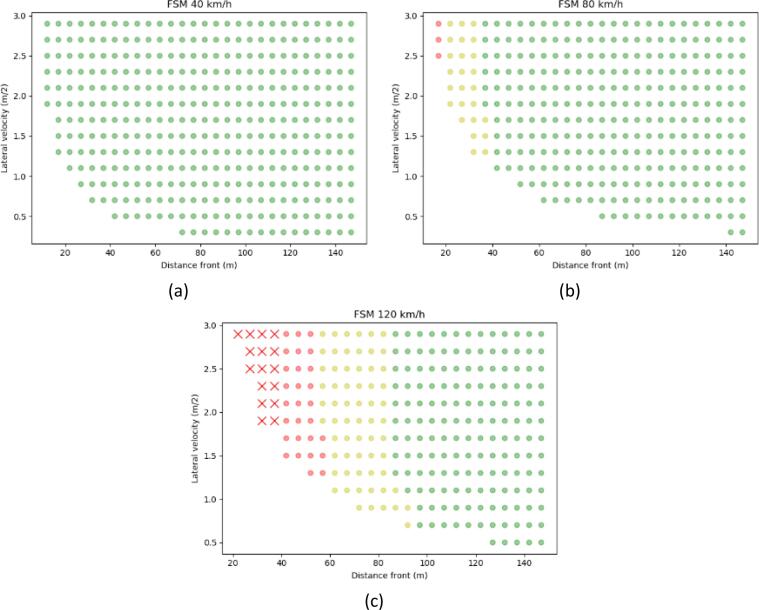
Classification of easy (green), medium (yellow), and difficult (red) scenarios according to the FSM level and crashes (“x”) for cut-out cases, for ego vehicle speed of (a) 40 km/h, (b) 80 km/h, (c) 130 km/h.

An example is shown in Fig. 7. The green dots represent the easiest cases and yellow are the medium difficulty cases. The most challenging preventable cases are shown by red dots, while the unpreventable cases when the simulated ego vehicle crashed into the object are denoted by red “x” markers. The ego vehicle speed and the distance to the static object are the most significant factors. The results for the rest of the cases are presented in this paper’s annex.

### 5.4 Validation: Cut-in cases

**Fig. 8:**
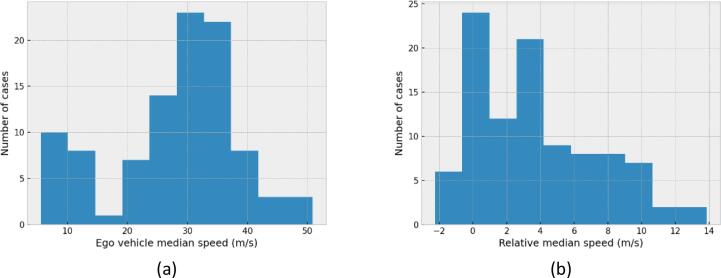
Histogram of (a) the median speed of the ego vehicle and (b) the relative median speed of the two vehicles, for the 99 most severe cut-in cases identified.

Overall, 99 cases of severe cut-in maneuvers have been identified in the highD dataset. As shown in the histograms of Fig. 8a, the median velocity of the ego vehicle covers a wide range, including low speeds and high speeds that are over the speed limit. Similarly, the relative velocity is presented in Fig. 8b, which is shown to be lower than 10 m/s for most cases.

**Fig. 9:**
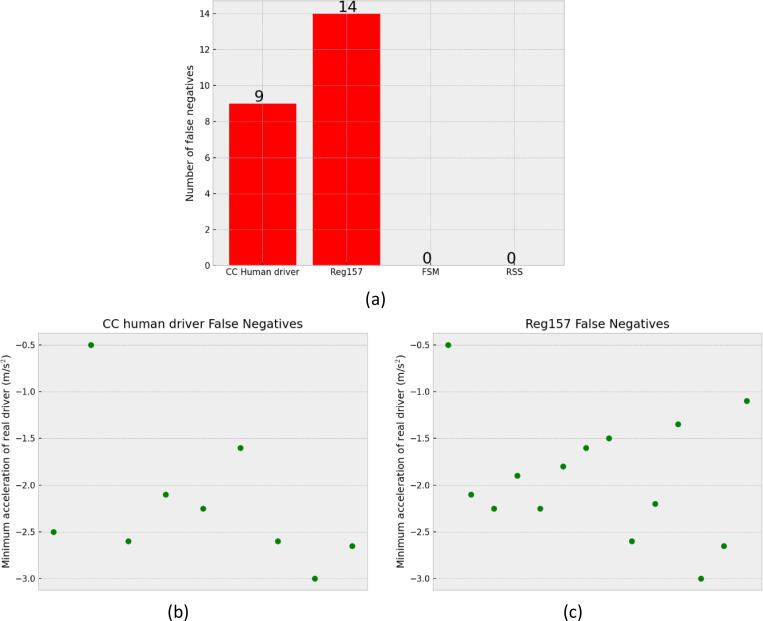
(a). Number of false negatives for each model. Minimum acceleration used for the human driver in false-positive cases of the (b) CC human driver model (c) Reg157 model.

Out of the 99 cases isolated, the RSS and the FSM are the only ones that classified all cases correctly as preventable for a human driver. Finding no false negative cases, i.e., cases incorrectly classified as unpreventable, is important for such a model in this specific use. The CC human driver model and the Req157 produced 9 and 14 false negatives respectively. The results are presented in the bar plot of Fig. 9a.

Models allowing collisions for situations in which a human driver would avoid a collision, could allow for automated driving systems that are not as safe as human drivers. Especially since the cases are based on real data so they occur in real traffic. The introduction of such systems to the market could increase the frequency of such accidents. Moreover, for all the cases that the CC human driver model and the Reg157 found to be unavoidable, the human drivers tracked in the dataset managed to avoid accidents without exploiting the full vehicle dynamics. The minimum acceleration value achieved for each case is presented in Fig. 9b and c for the CC human driver model and the Reg157 model respectively. For all the cases in which those models identified an unavoidable collision, the human driver never exceeded a deceleration of 3 m/s^2^. Moreover, during the simulations of the FSM for the same cases, the accident was avoided without the use of decelerations harder than 4 m/s^2^. This further shows the importance of the “tactical” safety level, with the driver anticipating emergencies before they become imminent.

### 5.5 Validation: Vehicles traveling in adjacent lanes cases

The number of vehicle pairs identified has been 243,897. For each case, the upstream vehicle, which is considered to be the ego vehicle, may have used some deceleration, either because of the movement of the other vehicle or as a result of the surrounding traffic. Regardless of the behavior of the tracked human driver, any deceleration requested by any of the models is considered to be a false positive, as the vehicle in the adjacent lane never crossed the lane markings.

**Fig. 10:**
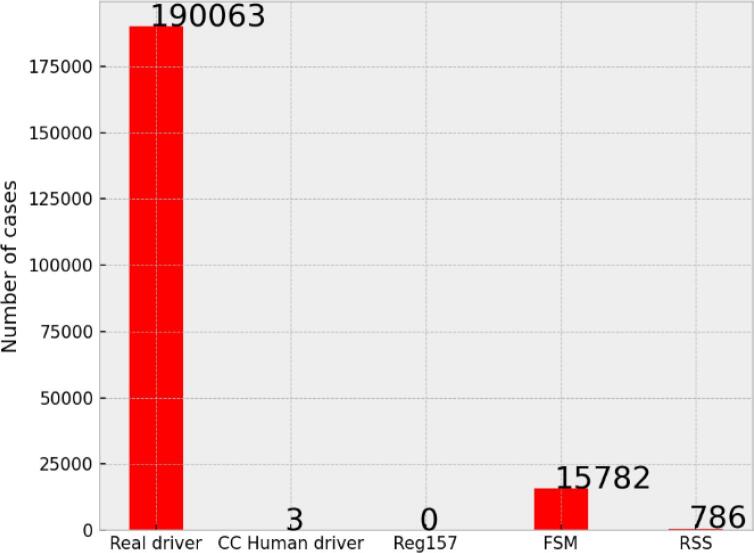
Number of cases where a deceleration was observed for each model and for the real drivers.

During the simulations, the Reg157 model was never activated, as the other vehicle never reached the lane markings. The CC human driver model was activated 3 times in total, showing a very small number of false positives. As presented in Fig. 10, the number of cases in which a deceleration was requested was 15,782 for the FSM model and 786 for the RSS-based model. This first finding indicates the FSM model is prone to false-positive classification, for the current parameter values. However, the actual vehicle had at least one instant of deceleration for 190,063 of the cases. For the human driver, we cannot assume that this deceleration is due to the other vehicle under investigation, as it could be caused by other reasons such as the surrounding traffic.

**Fig. 11:**
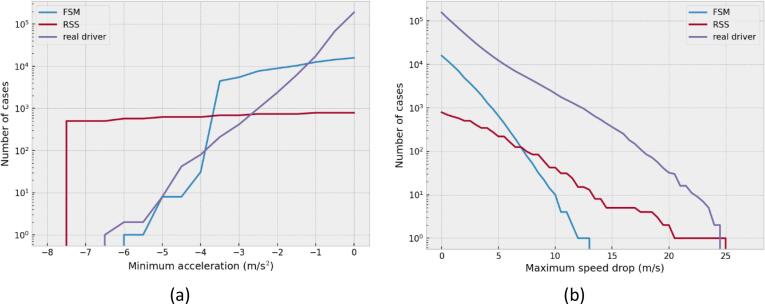
Cumulative distribution of (a) maximum deceleration value (b) maximum speed drop.

A more informative illustration of the results is presented in Fig. 11a and b. In Fig. 11a the number of vehicles achieving a minimum deceleration value is shown for a range of deceleration values, on a logarithmic scale for better readability. The capability of the FSM to judge the safety level based on fuzzy metrics and react according to the level of unsafety of the situation creates a large gap between the results of FSM and RSS. While the RSS model decelerated in much fewer cases, the deceleration has been much harder. Those kinds of maneuvers can affect both traffic flow and traffic safety. It seems it is very different from a human driver’s behavior, so that would probably be a surprising behavior for the surrounding traffic. Setting different parameter values would affect the magnitude of the deceleration for the RSS, but the shape of the distribution is not expected to change. This is because the reaction of the controller is binary (active or inactive), which comes from using crisp sets for describing the safety conditions.

On the contrary, the FSM decelerated for more cases than any other model. However, the cumulative distribution plot of the minimum deceleration shows how this is not dissimilar to the behavior of the human driver. The larger difference is shown in the range from −4 m/s^2^ to −2 m/s^2^ that are the boundaries of a comfortable deceleration that have been set. The parameter values have not been optimized to fit the results. It is shown how the FSM distribution is the only one that has the capacity to track, with some error, the real distribution. Moreover, for the proposed parameter values, the cases of hard deceleration with the FSM are even less than the ones recorded for the human drivers. This suggests that the decelerations assumed would not be outside what is common or frequent in the real world and they would not surprise the surrounding traffic.

Apart from the maximum deceleration value achieved, which represents the maximum intensity of the deceleration, the duration plays an important role. To show this, the maximum speed drop from the initial speed is presented in Fig. 11b for the observed data, the FSM, and the RSS model. The empirical cumulative distribution of the actual human driver is higher than both models. The only exception is an outlier of the RSS model with a speed reduction of 25 m/s. Those results indicate that FSM would not request very hard decelerations with a high duration. Moreover, it would not deteriorate the flow by requesting large speed drops. It has to be clarified that any manufacturer could use any control strategy they would like, as far as it is shown to be as safe as the reference model. Therefore, a manufacturer, having more accurate knowledge of the response time and the vehicle dynamics, could produce automated driving systems that are as safe, with an even better impact on the traffic flow. Especially considering that the response time of an automated driving system could potentially be shorter than the 0.75 s assumed for the FSM.

## 6 Conclusions

Automated driving systems bring important promises to the future of road transportation. Increased safety and efficiency, decreased environmental impacts, and time in congestion are some of the possible significant benefits. Meanwhile, more equitable access to transportation and overall better use of the infrastructure could, in the long run, bring large societal benefits. For all those to be realized, the ADS should be accepted by the public. Safety is a crucial precondition for the public’s trust, and for the systems to become dominant in the market. However, ensuring safety is not a trivial task. Regulations can be important on this path, and an example is the first global regulation on the approval of a Level 3 ADS adopted in 2021.

In the present paper, we present and explore the models used in the existing regulation to set safety performance requirements and additional two that were proposed in the regulation amendment, namely, the RSS, an industry proposed safety framework, and the FSM proposed by the authors.

The analysis shows that the models included in the existing regulation only focus on the reaction of the ADS to emergencies (similarly to what would be appropriate for ADAS systems). On the other hand, some of the field experts suggest that ADS should be able to drive defensively, predicting and avoiding emergencies in advance, as this is an important skill of an expert driver. An obvious trade-off exists, as the strictest regulations can have impacts similar to that of operation requirements, while less restrictive ones may not properly embody the ambitions for safe automated traffic.

Regarding the RSS model, while this is not the main scope of the model developers, interesting comparisons can be drawn between the performance requirements produced by it and the existing regulation. RSS can shrink the set of unpreventable cases. On the other hand, some cases that would not lead to an accident if no reaction was required, resulted in accidents when using the RSS model. Finally, the model proposed by the authors, based on fuzzy surrogate safety metrics, is able to simulate a defensive driving behavior and includes the ability of a calm, proactive reaction. The unpreventable cases identified are qualitatively close to an intersection between the other three models.

It is shown that in case anticipative driving is requested, the proposed model, FSM, can be used to derive the sets of preventable or unpreventable cases. This would not restrict the ADS operation, as it represents a minimum level, and the way of reacting is not dictated. Thus, such a model would not delay the potential traffic flow and efficiency benefits. Moreover, the proposed model can be used for the classification of preventable cases, according to the level of challenge. This can be very useful for the testing requirements, which can also be a part of such regulation.

Finally, the models have been validated for the cut-in case, using trajectory data from real drivers. It is shown that careful human drivers are able to proactively identify potential emergencies and use calm reactions to avoid them. Neglecting this crucial part, both the CC human driver model and the Reg157 model produced an unnecessarily large number of false-negative cases. This accounts for a non-optimal ambition in the actual efficiency of automated driving systems and could potentially lead to systems that are not as safe as a human driver reaching the public streets.

On the other hand, the investigation of false-positive cases has shown that FSM would request a deceleration for more cases than the RSS model. However, the requested deceleration would be milder, and the overall speed-drop smaller for the FSM. The RSS model is still based on a binary definition of what is considered to be safe and unsafe, so all reactions are requesting a very hard deceleration. FSM was shown to be better equipped to track the actual cumulative distribution of decelerations for human drivers. In the future the authors plan to validate on additional datasets, possibly coming from different regions, to further validate the model and the suitable parameter values.

For all the above, the FSM was successfully proposed by the authors to represent a new way to define the performance requirements of Level 3 motorway ADS in the amendment of UN Regulation 157.

### CRediT authorship contribution statement

**K. Mattas:** Conceptualization, Data curation, Methodology, Visualization, Formal analysis, Software, Writing – original draft, Writing – review & editing. **G. Albano:** Data curation, Formal analysis, Software. **R. Donà:** Formal analysis, Software, Writing – original draft, Writing – review & editing. **M.C. Galassi:** Funding acquisition, Project administration, Writing – original draft, Writing – review & editing. **R. Suarez-Bertoa:** Writing – original draft, Writing – review & editing. **S. Vass:** Writing – original draft, Writing – review & editing. **B. Ciuffo:** Conceptualization, Methodology, Visualization, Funding acquisition, Project administration, Writing – original draft, Writing – review & editing.

## Declaration of Competing Interest

The authors declare that they have no known competing financial interests or personal relationships that could have appeared to influence the work reported in this paper.

## Footnotes

1 Procrustes was a mythological bandit, who would put his captives on an iron bed that had the “ideal” height for a human. He would harm them in the effort of fitting them to that ideal height. While he has been slain by the mythological hero Theseus, the “procrustean bed” lives on, as an expression to describe arbitrary standards that bring harmful side effects.

2 github.com/ec-jrc/JRC-FSM.
